# Staff Members’ Experience of Italian Shelters for LGBTQIA+ Homeless and Runaway People: An Exploratory Study

**DOI:** 10.3390/ijerph20136214

**Published:** 2023-06-24

**Authors:** Elena Tubertini, Agostino Carbone, Massimo Santinello

**Affiliations:** 1 Department of Developmental Psychology and Socialisation, University of Padua, 35131 Padova, Italy; elena.tubertini@phd.unipd.it (E.T.); massimo.santinello@unipd.it (M.S.); 2 Department of Developmental and Social Psychology, Faculty of Medicine and Psychology, Sapienza University of Rome, 00185 Rome, Italy; 3 Department of Health Policy–The London School of Economics and Political Sciences, CPEC–Care Policy and Evaluation Center, St Clement’s Ln, London WC2A 2HD, UK

**Keywords:** LGBTQIA+, homeless services, southern Europe, psychological intervention, health psychology

## Abstract

Background: Some LGBTQIA+ people, after coming out, experience marginalization and homelessness due to rejection and discrimination from their family and community. The increase in support requests led to the creation of LGBTQIA+ temporary shelter homes worldwide. This study aims to explore the functioning and effectiveness of shelters, analyzing the experiences of staff members in Italy. Methods: Focus groups were held with a total of 15 staff members (age range: 32–53) working in three shelters for LGBTQIA+ people. Data were analyzed qualitatively through the grounded theory methodology. Results: Data coding showed five final core categories: (1) user characteristics; (2) staff characteristics; (3) community relations; (4) activities carried out by services; (5) criteria for intervention assessment and staff satisfaction. Results revealed some criticalities in the effectiveness of these services, particularly the difficulty in achieving autonomy for users, a weakness attributable to the non-exhaustive training of staff members and the funding discontinuity. Conclusion: To improve the efficacy of shelters, this study emphasizes the necessity to (a) carry out an analysis of the vulnerability of the local LGBTQIA+ community, (b) establish a stable network with local services (NHS system), and (c) implement staff members’ psychological training.

## 1. Introduction

Stigmatization, particularly toward sexual and gender minorities (SGM), increases the risk of being marginalized or becoming short- or long-term homeless [1,2]. This is especially true for lesbian, gay, bisexual, transgender, queer, intersexual, asexual, plus other identities (LGBTQIA+) individuals who face polyvictimization (LGBTQIA+ migrants, LGBTQIA+ youth, transgender women, and elderly or unemployed LGBTQIA+ adults) or for those who live in smaller urban communities and isolated rural areas [3,4,5,6]. In fact, evidence shows an overrepresentation of LGBTQIA+RH (LGBTQ+ people who run away from home or experience homelessness) within the general homeless population [3,4,7,8,9,10]. The reasons which can lead LGBTQIA+ people to a serious condition of marginalization and housing instability differ from those of heterosexual and cisgender people [4,11]. The main reason is rooted in the rejection of their sexual orientation or gender identity by their own family and parents [9,12]. This rejection may manifest violently, in the form of verbal or physical abuse by parents/guardians or forced expulsion from the household [1,13,14]. Furthermore, LGBTQIA+ marginalization is associated with prejudice and discrimination in other social environments, such as schools, workplace, health and social care, and foster care [15,16,17,18,19].

Once on the streets, being homeless or a runaway in unfriendly urban contexts can place LGBTQIA+ people more at risk for further victimization and vulnerabilities: deviant behavior, association with criminal organizations, violence, abduction, forced prostitution, risky sexual behavior, depression, suicidal ideation and attempts, and alcohol and drug abuse [20,21,22,23,24].

### 1.1. Historical Evolution of LGBTQ+ Self-Help and Cohousing Experiences

Over the years, the awareness of the abovementioned psychophysical risks and precarious conditions generated in the LGBTQIA+ community the need for service provision to their own members, in the forms of psychological support, solidarity, and cohabitation free of charge for the most vulnerable individuals. Historically, due to the sheltering and self-help attitude of the LGBTQIA+ community in the 1970s and 1980s at large [25,26,27,28], a complex array of networks, institutions, co-housing experiences, temporary recovery homes, and shelters began to emerge in large North American cities such as New York and San Francisco (alongside the already existing queer areas, generally recognized as places of congregation where LGBTQIA+ people could benefit from safe resources and services specifically aimed at the community) to provide hospitality to young LGBTQIA+ people who had run away from violent rural contexts [27]. The first private shelters were in Stonewall, often established by local queer associations renting out rooms to homeless or runaway LGBTQIA+ people [27,29,30].

Thanks to these experiences, there has been a surge in coming out over the years, even in the most marginal and non-inclusive contexts, leading to an increase in violence and victimization against LGBTQIA+ people within unsafe environments [20,24]. In response to this growing problem, various housing facilities (also referred to as “transitional housing”, “emergency shelters”, and “recovery houses”) have been established since the 2000s specifically for this population to promote their social rehabilitation and personal development [31]. These programs, often arising from volunteering experiences of local LGBTQIA+ associations, work with many types of LGBTQIA+ users and contexts, employing intervention models and values that may have applications in different areas (e.g., urban vs. rural districts). There are many examples of facilities for LGBTQIA+ homeless people in the United States [32,33,34,35]. These centers offer not only housing, but also a wide range of educational, social, and physical and mental health services to support marginalized LGBTQIA+ people in all their specific needs, with the aim of leading them to self-sufficiency. Activities vary from skills development workshops to employment and educational assistance, HIV testing, free primary healthcare and psychiatric services, care of elderly LGBTQIA+ people, and legal support for transgender people and LGBTQIA+ asylum seekers. Recent studies show that most users (42%) address a service specifically because of the presence of openly supportive staff or personnel belonging to the LGBTQIA+ community.

In Canada, the first transitional housing programs that aimed to provide safe and affirming spaces for LGBTQIA+ people were founded in Toronto in 2016 (YMCA Sprott House and the Friends of Ruby Home, Toronto, Canada), followed by many others; these projects offer activities, trainings, vocational mentoring, and mental and physical health services [35,36,37].

Services exist in South America providing shelter and healthcare for LGBTQIA+ people victims of violence, LGBTQIA+ migrants fleeing persecution, and young people who are expelled from their homes because of their sexual orientation, gender identity, and/or gender expression [38,39,40,41].

For what concerns Europe, some examples of co-housing experiences for LGBTQIA+ homeless youth (in Slovenia, Albania, and Italy) were collected in the report “17 Practices to help end Youth Homelessness in Europe” [42]. Some specific intervention models, such as Housing First, are expected to be beneficial for organisations working with LGBTQIA+ homeless people in Europe [43].

### 1.2. LGBTQ+ Recovery Housing Experience: State of Art

Studies have been carried out to address the growing problem of homeless, runaway and marginalized LGBTQIA+ people, coupled with the lack of specific services, to identify existing targeted programs for this population, especially in North America and Canada [4,8]. These studies were designed to determine the characteristics and the efficacy of the interventions, to guide future practice, policy, and research with LGBTQIA+ runaway or homeless people [27,37] A review by Ferguson and Maccio [4] shows that the strengths of the evaluated housing programs for runaway and homeless LGBTQIA+ youth could be summarized by five main components: (a) reliance on evidence-based health and psychological research; (b) attention to the mental health of the users and their exposure to past traumatic events; (c) provision of a safe, stable, and supportive housing environment; (d) the employment of staff from the LGBTQIA+ community; (e) reciprocity and networking between LGBTQIA+ runaway homeless youth and cisgender/heterosexual peers. The literature also points to several weaknesses, including the fact that existing programs for the general population of homeless or runaway people (not specifically targeted for gender or sexual minority people) usually do not have all the abovementioned features. Often, emergency shelters, which welcome any homeless person regardless of their gender or sexual orientation, do not represent a safe space for LGBTQIA+ people because of the occurrence of bullying and discrimination, heterocisnormativity, improper use of pronouns, incorrect gendered room assignment, and violence [44]. To address these issues, research on policy implications has been carried on facilitating the development of homeless-serving programs inclusive and affirming of transgender people [45]. Nevertheless, the scientific literature is still lacking research on this specific target and interventions, partly because data on sexual orientation or gender identity are rarely collected by emergency shelters, and LGBTQIA+ people are often unwilling to disclose their identities in these contexts.

## 2. Current Research

The study is aimed at filling the gap in the literature concerning studies on LGBTQIA+ housing services in the Italian context, a developing and emerging reality within the framework of social policies addressing the LGBTQIA+ community. The goal is to develop policy recommendations to support services and further research on this topic. The specific objectives of this study are (a) exploring the characteristics and roles of the staff and the organizational functioning, reception methods, work models, and activities offered by the services, (b) exploring criteria for the interventions’ assessment, feedback, and sources of satisfaction of the staff, starting from their fieldwork experience, and (c) suggesting practical implications for shelters, service providers, and policymakers to respond to the needs of this population and to enhance knowledge and research in this field.

## 3. Materials and Methods

### 3.1. Participants

Participants were 15 staff members (seven men, eight women; age range 32–53) of three main Italian housing facilities for marginalized, runaway, or homeless LGBTQIA+ people. Table 1 shows the characteristics of the housing facilities involved in the research. Most of the staff members interviewed stated to belong to the LGBTQIA+ community. Participants were recruited according to a snowball-like sampling strategy among the staff of the three Italian housing facilities. Table 2 reports the total number of staff members working in the service, as well as their roles and tasks. In bold are highlighted the professions that were represented in the focus groups. Participants coming from the same facilities had been working together for three or more years and, therefore, knew each other beforehand. All focus groups were attended, for each service, by at least two founding members of the housing projects, who contributed to the planning phase of the services.

### 3.2. Instrument for Data Gathering

The research was conducted through the focus group method to collect participants’ opinions and attitudes on their fieldwork experience in the Italian LGBTQIA+ housing facilities. The questions for the focus groups were created by the research team, consisting of one junior researcher, one researcher, and a senior professorial researcher. The focus groups were conducted on the basis of guidance questions focusing on (1) working time and role in the service; major types of users welcomed by the service, (2) service activities and interventions considered most effective, and (3) main sources of satisfaction in their work. To draft the questions and decide on which aspects were to be explored, it was important to have an overview of the literature and knowledge already acquired on the main dimensions of the phenomenon. Although a consistent interview guide was developed and used for all sessions, some changes to the outline had to be made during the process. Following the “spiral” approach [46], on the basis of the feedback collected during the first focus group, the questions were articulated in a more targeted and clearer way.

### 3.3. Procedures

Data were collected between March and May 2021. A total of three (*n* = 3) focus groups were carried out, one for each recovery house, formed by four, five, and six participants, respectively. Each focus group took 90–120 min to complete. The interviews were conducted online by the principal investigator. Due to the COVID-19 pandemic, it was necessary to involve participants only through virtual means of communication. It was judged preferable to schedule only one meeting per structure; doing so allowed all participants to attend at one time. Informed consent was obtained from all participants through an online form. All procedures performed with human participants were conducted following the ethical standards of the 1964 Helsinki Declaration, of Italian Psychology Association AIP and approved by the ethical committee of the Department of Developmental and Social Psychology of Padua University (prot. n. 2487).

## 4. Data Analysis

The focus groups’ recordings were transcribed confidentially, preserving participants’ privacy about their personal data, and replacing their first and last names with random letters. A grounded theory analysis was carried out without the use of software, using a coding [47] low technology “scissors and sort” method [48,49,50]; this method consists of cutting up the transcripts found to be conceptually similar or related in meaning utilizing color markers and grouping them into categories. The steps of analyzing focus group data are coding, categorizing, and making intergroup comparisons. In the first instance, the text was reread, and then a classification system was constructed by matching a color to each category and subcategory corresponding to the main topics raised during the sessions. Subsequently, the parts of the text corresponding to the identified categories were highlighted with marking pens in the corresponding color; these parts of the text could be concepts, interactions, single sentences, or whole passages. Lastly, the parts corresponding to the same category were grouped together to make the contents easily comparable. During transcription, nonverbal communication (voice tone, speaking turns, laughter, and gestures) was considered if salient for understanding group dynamics and interactions.

The categories were identified by the principal investigator and then redefined and discussed with the rest of the research team. It was considered appropriate to proceed with a double-blind analysis of the content of the focus groups. The identified categories and the different interpretations were then compared to agree on common results. Following the analysis, the results were discussed and reviewed with some operators and stakeholders and potential users involved in promoting LBGTQIA+ policies.

## 5. Results

The following are the main findings, divided according to the six core categories and respective subcategories identified in the analysis: (1) user characteristics; (2) staff characteristics; (3) networks with community; (4) activities carried out by the services; (5) criteria for the intervention assessment. Table 3 gives an overview of categories and subcategories. Notable parts of the interviews’ answers are reported in Table 4.

### 5.1. User Characteristics

Participants identified transgender people as the main users of the provided services [51,52], followed in order by gay, lesbian, and bisexual people, and people with other sexual orientations and all other queer identities.

Regarding age, it emerged from the focus groups that, in most cases, the services host young people between 18 and 25 years old; in no case are minors under the age of 18 hosted (see Table 4). Participants also indicated that the age of users has decreased since the services were opened. It should be noted that, in some cases, the facilities were only aimed at young people, while one facility included LGBTQIA+ elderly people among the possible users of the project. As for the motivations for leaving the household, it emerged from the participants’ words (see Table 4) that runaway people (those who seek shelter after running away from home) had in common an experience of traumatic rejection within dysfunctional family dynamics following their coming out, manifested in the form of violence (on themselves or by their parents); this rejection was often determined by religious motivations and can occur in families with different sociocultural backgrounds. Other users were either homeless or severely marginalized because of their gender identity or sexual orientation, and most of them were trans people (who experienced living on the street, extreme poverty, and forced prostitution) who needed to register their residence on their identity documents to start their transition.

A particularly relevant situation according to the participants within the facilities involved foreign LGBTQIA+ people, asylum seekers or refugees, users with different comorbidities in terms of mental disorders and psychiatric pathologies, and even cases of self-harm or attempted suicide because of discrimination. In some instances, LGBTQIA+ users with disabilities were hosted. Cases in which the guests were driven to substance abuse were frequent but not all facilities allowed them admission and treatment if they still used drugs. Table 4 presents some examples of cases that emerged from the interviews.

### 5.2. Staff Characteristics

Often, staff members were not employed by the housing structure, but worked in another context and collaborated on a voluntary and unpaid basis within the services. In some cases, a clear dividing line emerged from the participants’ words between the role of staff and that of non-specialist volunteers, i.e., those who were part of the associations as LGBTQIA+ activists even before the housing services were set up (see Table 4); this division sometimes proved problematic. Tasks appeared to be distributed according to each person’s professional knowledge. Only in one of the structures did the participants state that roles were not always dedicated, and skills were limited to experience and self-training in the field.

### 5.3. Networks with Community

With regard to the network between LGBTQIA+ housing facilities and the external community, three subcategories were identified.

- Local network with institutions

Participants reported that, in some cases, collaboration with the local institutions (e.g., municipalities) was limited to projects already started with previous fundings; in other cases, the municipality took over the co-management of the flats, and the facilities were only partially maintained thanks to donations. All facilities could count on a wide network and many partnerships, which allowed them to redirect cases that one facility cannot accommodate to another. Collaborations with companies, schools, or other public or private services, as well as staff training on LGBTQIA+ issues and inclusiveness, were also important for practitioners (see Table 4 for examples).

b. Media network

The help requests not only came from the local area but were also from outside the province, either self-reported (through the Internet and social media) or made by third parties (family members, friends, associations, and public bodies) who could easily reach the facilities via contacts published on their website and social networks.

c. Informal network

From the focus groups, the existence of a strong informal network emerged, which allowed emergency situations to be identified. Some examples were reports coming from friends or associations with which the structures had a long history of collaboration.

### 5.4. Activities Carried out by the Services

Common to all facilities was the presence of a help desk service, which handled reports, filtered requests, and directed those who could not be accommodated to other projects or existing organizations in the area. In the event of emergencies requiring priority intervention, placement as guests in the housing services was arranged. The first operational phases of the reception highlighted by the focus groups participants were aimed at understanding the needs and expectations of the user at a psychological and residential level (with respect to the length of stay in the facility, the type of educational and clinical pathway, etc.).

After the reception, weekly medium-term objectives were identified with the users, described by the staff as daily goals and priorities linked to personal and relational autonomy. Examples of medium-term objectives were maintaining a collaborative climate with other guests, involvement in social community activities, building trust and clinical compliance, and autonomy in structuring practical aspects of daily life (e.g., shopping, cooking, and cleaning).

There were also long-term objectives, resulting from a preliminary needs analysis carried out at the beginning of the placement. These goals were described by the staff as “the results that the person intends to achieve during their stay in the facility”, and they were established by the users in order to achieve autonomy on several levels (such as school, work, economic, housing, and legal level), personal independence, and psychological and relational stability (escape from violent family situations or reconciling with their families).

### 5.5. Criteria for the Interventions’ Assessment

Identifying clear indicators of the project’s effectiveness was complex for the participants, who agreed that, in a short period of time (about 8 months), achieving the pre-established objective of complete independence was too ambitious to be the only indicator of effectiveness. Staff members identified seven subcategories among the intervention practices implemented by different services: personalization of objectives (a), competence of the team (b), sensitivity of the territory (c), quality of the network (d), definition and selection of users (e), structuring of a welcoming environment (f), and staff satisfaction (g) (see Table 4 for quotations).

Staff members reported that, to be effective and to allow the user to reach awareness about their condition and plan their future, the objectives had to be personalized, consistent with needs and available resources, flexible, and adaptable over time. Fundamental to this process, according to the participants, was the synergy among different professionals, the multidisciplinary approach and the team cohesion (see Table 4 for some examples of answers).

Participants also believed that the inclusiveness of their communities and territories (regions, municipalities, etc.) about LGBTQIA+ issues was indicative of effectiveness of the program (see Table 4). The presence of a quality network, involving different resources and public and private bodies, was reported as essential to achieving personalized objectives.

Even within the facilities themselves, staff members identified setting up a welcoming environment, recalling the concept of “home”, as an effective practice.

The selection of users within a restricted age range seemed to facilitate a positive climate among peers who shared similar needs and experiences.

Moreover, it emerged from the staff’s answers that filtering requests, by limiting access to people with serious psychiatric disorders or addictions, was effective in preserving pre-existing ties between users or with staff.

Ultimately, satisfaction was identified by participants as a complex construct, not directly related to service effectiveness, and which could not be taken as a single indicator for evaluation. Satisfaction could depend on the helping dimension of the profession (subjective, internal experience) or on external factors. It emerged from the answers that, in the experience of the practitioners, satisfaction could be linked both to feedback from the user (e.g., arriving on time at the session, achieving an objective, and establishing positive relationships) and to the positive relationship with colleagues, the network, partners, and the social system.

For staff members who themselves belonged to the LGBTQIA+ community, the dimension of satisfaction was linked to queer generativity [51,53], meaning the possibility of giving back the personal, relational, and social support that was given to them in the past to other people from the LGBTQIA+ community in their most dire moment of need (see Table 4).

## 6. Discussions

The results highlight the need for further investigation of some of the issues raised by the staff members in relation to the social mandate of the facilities, the users, the objectives underlying their professional role, and the criteria for monitoring the activities of these services.

LGBTQIA+ runaway or homeless people are characterized by type and severity of health risks, in accordance with the scientific literature [8,12,54]. Users who encounter the services of the analyzed facilities reflect only a part of the LGBTQIA+ runaway and homeless community. This population stands out because it finds in these services a point of contact which is attentive to its needs, sometimes resulting from the sum of multiple aspects (violence, homelessness, forced prostitution, LGBTQIA+ people with disabilities, transgender immigrants or refugees, and LGBTQIA+ elderly people in loneliness or extreme poverty). It is noteworthy that transgender people account for the largest proportion of users of these services, as they experience the greatest stigma and discomfort in the contexts to which they belong (family of origin, neighborhood, and friendships). On the other hand, it also suggests that these people find better understanding and help for their condition in these targeted structures, in contrast to other services offered by the national health or welfare system. [55,56]. Moreover, the respondents also defined that a considerable part of the users is composed of people with a psychiatric diagnosis suffering from addictions or with a double diagnosis. Individuals turning to the services solely for problems connected to their sexual orientation, such as the nonacceptance of coming out by the family context of origin [57], constitute a minority. This result surprised the expectations of the promoters of these services, because, when they first planned the service, they imagined a target group consisting mainly of non-heterosexual people (without particularly complex problems). Results showed that the target group is mainly composed of people with different levels of vulnerability and severe adaptation problems. This has increased the difficulties of the staff in proposing services that are up to the task of rehabilitating users at a social level and has created situations of noncompliance, compromising the achievement of long-term objectives. These experiences suggest the need to rethink the target of services and the professional competences needed to take care of the most fragile users’ needs.

Results revealed a problematic issue regarding the confusing relationships between the service provider and the staff. The latter go from being volunteers in the associations which promote the projects to being practitioners in charge of professional roles within the housing services, which has several consequences. Firstly, in line with scientific studies, the staff sometimes lacks the necessary preparation to deal with LGBTQIA+ marginalization situations, potentially creating discomfort among the users, who may feel misunderstood or unsafe [12,58], extending their homeless status [59]. Secondly, the discrepancy between intrinsic motivation (queer/activist identity, community belonging, and queer generativity) and extrinsic motivation (role of health professional within the service) can generate confusion for both users and staff, who may not be able to self-identify within a professional framework, but rather prioritize their role as activists. This can make it difficult for staff to build a training pathway for the acquisition of professional skills, given the job discontinuity allowed by the projects (calls and public grants) that sustain the services. This underlines the need for highly qualified staff and for the presence of psychologists, psychiatrists, social educators, cultural mediators, and health and social policy advisors/consultants within the team, for whom continuous trainings on the topic of care for LGBTQIA+ people are necessary. Lastly, the lack of staff in terms of numbers sometimes makes the presence of volunteers essential.

A certainly useful dimension to be discussed is the relationship between the LGBTQIA+ housing facilities and the local health services, the surrounding community, and the cultural media-shaped aspects. The relationship with the public health services is discontinuous and still under construction. It seems essential, given the care of a fragile group of users (previously discussed), to establish an integrative external care system (e.g., joint work with operators of the mental health centers, psychiatric services, or centers for addictions treatment). This difficulty could be caused by the fact that the professional staff members who work in these facilities are not informed about the social mandate of the health services, and that those national services may not be able to find suitable interlocutors in the LGBTQIA+ shelters, since they are not part of the National Health System. On the other hand, shelters’ staff may not identify LGBTQIA+ people as possible users of public health services, since they do not believe that general public health services (not targeted for LGBTQIA+ people) are sufficiently competent in caring for sexual and gender minorities, as often found in the literature [56].

Concerning the collaboration with the associations within the local context, all the facilities appear to be well established within their territory, counting on a wide network. The possibility of developing a service of this kind is also related to the fact that all analyzed projects are located in large urban centers with a large student population and a well-rooted history of the LGBTQIA+ movement. In fact, it is important to underline that these areas are important centers with regard to media communication (cinema, fashion, magazines, and radio), LGBTQIA+ meeting venues (clubs, bookshops, and arci circles), university degree courses on gender issues, LGBTQIA+-friendly associations and administrations, and community services. Despite Italy’s in-between situation with regard to the controversial positions of the catholic church and legislative aspects (the law for civil unions does not include stepchild adoptions, and the bill which extended the categories of anti-discrimination law to sexual orientation, gender identity, and disability, also known as the “Zan bill” [60], was rejected by the Italian Senate in November 2021), the presence of this LGBTQIA+ culture within the social context and formal and informal collaborations can be considered a positive factor that allows these cities to be recognized as places of refuge for people living in surrounding areas [61]. This makes it possible to improve the monitoring of LGBTQIA+ people’s needs in the territory.

At the same time, a good network allows for the redirection of cases that the facilities are not able to welcome to other types of services. It turns out that the expectations of the guests do not always correspond to what the service can offer. In these cases, it is essential to clarify the type of users that can be accommodated, establishing criteria designed according to the specific context and the available competences.

An important aspect of the work carried out in these shelters is the construction, achievement, and assessment of objectives in relation to the users’ requests. Goals such as the users’ wellbeing and autonomy are difficult to achieve in a social rehabilitation intervention for services that operate in emergency situations and with a short intake period. These goals, due to the time and effort they require, can hardly ever represent a useful parameter to evaluate the effectiveness of the project for a vulnerable user (as highlighted above). However, short- and mid-term objectives (e.g., exiting violent family situations, and reducing risky or self-damaging behaviors) can be taken into consideration for assessing effectiveness. Staff members believe that, to achieve these goals, a process of personalization of the programs is necessary, aiming at the greatest possible flexibility in remodeling the objectives according to the type of users and their needs. The criticality of this approach lies in the fact that it is not always possible for practitioners to consider both the time limits and the users’ personal resources. As pointed out by the participants, the needs of the users and the objectives cannot be defined a priori, as this would risk addressing, according to the participants, the urges of the person, without corresponding to the individual’s real needs within a psychological dimension.

Another element of feedback emerging from the discussion with the staff is the satisfaction dimension. This can represent an internal evaluation criterion concerning the experiences of the services’ staff. It appears relevant that the sources of satisfaction for the interviewed practitioners reside mainly in the quality of their relationships with the users and in the dimension of queer generativity [51,53]. Belonging to the LGBTQIA+ community seems in fact to represent for the staff a way in which an identity aspect is transformed into a prosocial dimension, giving back to the community the help and the personal, relational, and social support received in the past. This reflects the empowerment aspect of the LGBTQIA+ community, also evident historically in the construction of community shelters starting from the community’s resources and solidarity [27,53]. In line with the literature, in fact, many individuals who seek out, create, or support LGBTQIA+ communities are seeking not only sociopolitical change [62,63], but also a fulfilment of internal needs: giving meaning to one’s personal dimension, finding social outlets, redeeming one’s invisibility and the discriminations experienced, and exercising freedom of expression [64], which are sources of gratification for the staff. This can, on the one hand, be considered a positive factor, since, in line with the scientific literature, several users contact certain centers specifically for the presence of openly supportive and friendly staff, or staff members who belong to the LGBTQIA+ community themselves [22]. However, it can sometimes be difficult for these practitioners to build up a care professionalism, when joining association activities is the result of an internal need for compensation.

## 7. Limitations of the Study and Future Directions

This paper had some limitations. The first one concerns the size of the group of participants in the focus groups, which was influenced by the COVID-19 pandemic, and which made it necessary to involve them only through virtual means of communication. In fact, there was no prior relationship between the research team and these centers. Another weakness concerns the data collection tool; the focus groups were all conducted online, which caused a decrease in engagement and interaction within the group, as well as disadvantaging the nonverbal dimension of the interactions.

A further limitation is given by the fact that the services’ users were not included as participants. Future research will necessarily have to include other housing experiences from different countries and involve the users of these services to evaluate their effectiveness according to their personal experiences. This study can serve as preliminary research for future projects, ideally aiming at assessing the social support and wellbeing of people in LGBTQIA+ homeless services, and investigating their community connectedness. Future in-depth studies of this type may be useful to understand which services’ characteristics positively influence users’ wellbeing, thus creating guidelines and questioning representatives of other institutions and bodies consistent with the development of LGBTQIA+ inclusive policies.

## 8. Policy Implications

In relation to the results presented above, it was considered extremely useful to provide guidelines and policy suggestions aimed at the development of good practices for the development, strengthening, and replicability of LGBTQIA+ reception and housing services.

Firstly, since it is not always clear what the social mandate of the service is, at a preliminary stage, an in-depth study of the territory and the needs of potential users is necessary to identify the function of the services within the context. Analyzing the relationship between how the facility plans to operate and how users foresee the type of help they will receive allows services to be better prepared and more competent in the provision of specific care and human resources.

The second point includes, during the first phases of the construction of the service, the training of staff members on the need analysis [65], which would allow the construction of appropriate settings, the understanding of the user who comes to the service, and the referral of the user to other services; this is possible through integrated care or continuum of care (CoC), which has been shown to be successful in meeting the immediate needs of the population served, supporting organizations to provide competent services to LGBTQIA+ [66]. Concerning the psychological intake and the management of clinical interviews, this competence appears fundamental for the construction of personalized objectives and is in line with literature that states the importance of training and supervision for social service providers’ work engagement and high-quality care [67].

Thirdly, setting up ongoing monitoring systems on users and staff with clear indicators (e.g., number of accesses and type of users, user satisfaction, organizational climate, and goal achievement) is essential to check the progress of the projects. Longitudinal studies after exiting the service are also important to verify the social rehabilitation of the users (e.g., number of people who have been reintegrated into their households, who have found a job and/or have achieved residential independence). Monitoring tools can be both qualitative and quantitative (e.g., focus groups, questionnaires, and interviews) and tailored according to the area to be monitored. Adequate monitoring would allow the service to adjust its activities and intervene to address some emerging criticalities.

While this research was in progress, in May 2021, the Ministry of Equal Opportunities and Family and UNAR (focal point of the national Equality Body) launched a call for proposals to promote and finance (with a budget of 4,000,000 EUR) the creation and dissemination of 37 free LGBTQIA+ services and residential facilities on the national territory [68].

This underlines the relevance and usability of this research and adds useful evidence to advance the literature on the topic [69].

## 9. Conclusions

This study was aimed at exploring the functioning and effectiveness of Italian shelters for homeless LGBTQIA+ people. Focus groups were held online with staff members working in three Italian shelters for LGBTQIA+ people. Data coding resulted in five core categories: (1) user characteristics; (2) staff characteristics; (3) community relations; (4) activities carried out by services; (5) criteria for the intervention assessment and staff satisfaction. Results revealed the difficulty in achieving autonomy for LGBTQIA+ service users, a weakness attributable to the non-exhaustive training of staff members and the funding discontinuity. To improve the efficacy of shelters, this study emphasizes the necessity to (a) carry out an analysis of the vulnerability of the local LGBTQIA+ community, (b) establish a stable network with local services (NHS system), and (c) implement staff members’ psychological training. These indications could be useful to national and European institutions or third-sector organizations for the construction of LGBTQIA+ housing projects.

Research on this topic is lacking in Europe, and this was the first study of its kind in Italy. Hopefully, this will open new research interest on the topic, given the rise in anti-LGBTQIA+ policies and discourse around the world.

## Figures and Tables

**Table 1:** Characteristics of Italian LGBTQIA+ housing facilities involved in the research.

Name	Active Since	Beds	Designation	Target
Service (A)	01/2017	2	Shelter and first aid home	LGBTQIA+ youth (18–35, Italian and foreigner), rejected from family/home or runaway due to violence discrimination
Service (B)	07/2019	6 (2 homes)	Shelter and reception home	LGBTQIA+ youth who are discriminated against by families
Service (C)	12/2018	24 (5 homes)	Social co-housing	LGBTQIA+ youth (18–26) runaway or rejected/abused by their families; LGBTQIA+ migrants and refugees; LGBTQIA+ elderly in loneliness or poverty; marginalized transgender people

**Table 2:** Overview of professionals working in the services and involved participants.

Service	Professionals in the Service	Role and Tasks	Participants
Service (A)	**Service consultant** (2) **Chairperson** (1) **Treasurer and teacher** (1) **Communication expert** (1) Lawyer (1) Psychologist (2)	Consultancy General management Supply management, education Social promotion, networking Legal helpdesk Counseling service	5
Service (B)	**Project referee** (2) **Housing referee** (1) **Educator** (2) **Immigration expert** (1) **Helpdesk responsible** (1) Psychologist (1) Psychiatrist (1)	General management Housing management Educational interventions Reception of migrants/refugees External contacts, first interviews Intake, psychological support Diagnostic interview, guest filter	6
Service (C)	**Project referee** (1) **Professional educator** (1) **Psychotherapist** (2) **Social worker** (4) Psychiatrist (1)	General management Educational interventions Intake, psychological support Helpdesk responsible Diagnostic interview, guest filter	4

**Table 3:** Categories and subcategories emerging from data analysis.

Categories	Sub-Categories
(1) User characteristics	Age Sexual orientation and gender identity Comorbidities or other issues Reasons for using the service
(2) Staff characteristics	Voluntary or hired staff Professional figures working in the service
(3) Networks with community	Local network with institutions Media network Informal network
(4) Activities carried out by the services	General practices for welcoming users Long- and short-term objectives
(5) Criteria for the intervention assessment	Individualized objectives and flexibility The staff Rising context awareness: inclusive systems Formal and informal network User/target definition The concept of “home” Sources of staff satisfaction, queer generativity

**Table 4:** Categories and representative quotations.

Area	Representative Quotations
User characteristics	“We work with transgender teenagers; for them, it’s more difficult to get a job.” “There is no common factor: families of any religion, who don’t allow children to be free, or families with a rather high cultural background, who don’t accept their children...” “The interconnection between migration and SGM is complex; it intersects a series of values.” “LGBTQ+ victims of violence, both from non-EU and EU countries; some come to Italy as political refugees because of persecution in their own countries.”
Staff characteristics	“We are volunteers; we have other jobs.” “We gradually specialized in certain areas. We all have a minimum level of training […], we can deal with any temporary emergencies that may occur.”
Networks with community	“We created a good network with the municipality, and we managed, in one dormitory, to provide a gender-free room dedicated to trans people.” “We look for associations that operate in various fields, to build a support network for the specific situations.” “We didn’t build a ‘ghetto’ for LGBTQ+ people, quite the opposite; our work is to create a network with others who can get to know our world, bring them in.”
Activities carried out by the services	“We try to make a good analysis beforehand; we evaluate which kind of support they need, and then we build a personalized plan to dampen difficulties.” “We plan their future together with them, we try to understand what their life expectations are…” “We don’t create standardized projects based on what we think the person’s needs are.”
Criteria for intervention assessment	“A certain ability to reprogram and be flexible.” “A multidisciplinary […] and integrated perspective makes it so effective.” “To raise awareness, even in big companies… so, if you find a CV of a trans girl, you read it, you take that interview.” “I would evaluate a service as effective when the social system supporting us is trained, aware, and actually becomes inclusive.” “Even after some time, the fact that they call us, give us feedback on what they have achieved outside of our project, is certainly a source of gratification.” “The thing that gratifies me is the working group and the team: I don’t feel I can be as authentic in another context.” “The compensation with respect to your experience, your life… As a member of the LGBTQ+ community, I feel I must give back a piece of what has been done for me by the community itself.”

## Data Availability

Data are available upon request from the corresponding author.
